# Possible Arbovirus Found in Virome of *Melophagus ovinus*

**DOI:** 10.3390/v13122375

**Published:** 2021-11-26

**Authors:** Alexander G. Litov, Oxana A. Belova, Ivan S. Kholodilov, Magomed N. Gadzhikurbanov, Larissa V. Gmyl, Natalia D. Oorzhak, Anna A. Saryglar, Aydar A. Ishmukhametov, Galina G. Karganova

**Affiliations:** 1 Laboratory of Biology of Arboviruses, FSASI Chumakov Federal Scientific Center for Research and Development of Immune-and-Biological Products of RAS, 108819 Moscow, Russia; novosti-wxo@yandex.ru (A.G.L.); mikasusha@bk.ru (O.A.B.); ivan-kholodilov@bk.ru (I.S.K.); magomed_19@mail.ru (M.N.G.); lvgmyl@mail.ru (L.V.G.); 2 Department of Biology, Lomonosov Moscow State University, 119991 Moscow, Russia; 3 Infectious Disease Hospital, 667003 Kyzyl, Russia; natalia.oorzhak@yandex.ru (N.D.O.); anna_kyzyl@mail.ru (A.A.S.); 4 Institute for Translational Medicine and Biotechnology, Sechenov University, 119991 Moscow, Russia; ishmukhametov@chumakovs.su; 5 FSASI Chumakov Federal Scientific Center for Research and Development of Immune-and-Biological Products of RAS, 108819 Moscow, Russia

**Keywords:** *Melophagus ovinus*, metagenomics, virus, virome, Republic of Tuva, *Iflaviridae*, *Rhabdoviridae*, *Solemoviridae*, *Reoviridae*, *Sigmavirus*

## Abstract

Members of the Lipopteninae subfamily are blood-sucking ectoparasites of mammals. The sheep ked (*Melophagus ovinus*) is a widely distributed ectoparasite of sheep. It can be found in most sheep-rearing areas and can cause skin irritation, restlessness, anemia, weight loss and skin injuries. Various bacteria and some viruses have been detected in *M. ovinus*; however, the virome of this ked has never been studied using modern approaches. Here, we study the virome of *M. ovinus* collected in the Republic of Tuva, Russia. In our research, we were able to assemble full genomes for five novel viruses, related to the *Rhabdoviridae* (*Sigmavirus*)*, Iflaviridae*, *Reoviridae* and *Solemoviridae* families. Four viruses were found in all five of the studied pools, while one virus was found in two pools. Phylogenetically, all of the novel viruses clustered together with various recently described arthropod viruses. All the discovered viruses were tested on their ability to replicate in the mammalian porcine embryo kidney (PEK) cell line. Aksy-Durug Melophagus sigmavirus RNA was detected in the PEK cell line cultural supernate after the first, second and third passages. Such data imply that this virus might be able to replicate in mammalian cells, and thus, can be considered as a possible arbovirus.

## 1. Introduction

With the advances in the transcriptomic approach, the number of newly described viruses has increased dramatically [1,2,3], which has led to a breakthrough in our understanding of viruses’ biodiversity and evolution and has led us to rethink the existing virus systematics [4]. Many novel viruses were discovered in arthropods [1,2,3]. Viruses of arthropods are objects of special interest in virology since they can be vectors of the arboviruses, i.e., viruses that cycle between invertebrate and vertebrate hosts [5]. Moreover, arthropod viruses provide us with insights into viral evolution, host switching and virus pathogenicity. Blood-sucking invertebrates are hosts to many arboviruses, causing diseases of humans and domestic animals, and possess a great challenge to the healthcare system and the agricultural industry around the world [5]. While viromes of the well-established vector invertebrates, such as various species of mosquitoes [6,7,8] and ixodid ticks [9,10], are actively studied, other blood-sucking invertebrates, such as louse flies, receive less attention.

Louse flies of the Lipopteninae subfamily are blood-sucking ectoparasites of mammals. The sheep ked (*Melophagus ovinus*) has been widely distributed with sheep. It can be found in the most sheep-rearing areas, including Europe, Asia, North America, South Africa and Australia. Sheep are considered to be the main host for *M. ovinus*, but it can also be found in a number of other species [11], such as European bison [12], goats, dogs, rabbits and even occasionally, humans [11]. Sheep ked parasitism may be responsible for economic losses due to its effect on the livestock. It can be the cause of skin irritation, restlessness, anemia, weight loss and skin injuries (caused by the animal itself in an attempt to alleviate the itching) [11]. The annual economic impact of *M. ovinus* infestation on just two tanneries has been estimated at USD 1.6 million due to the decreased sheep skin quality [13].

Apart from the damage caused by the parasite itself, there is a discussion on the possible vector competence of *M. ovinus*. Various bacterial and protist species have been identified in sheep keds, including some that are pathogenic, such as *Borrelia burgdorferi* [14], *Anaplasma phagocytophilum* [15], *A. ovis* [15,16], *Bartonella melophagi* [17], *Theileria* spp. [18] and *Trypanosoma (Megatrypanum) melophagium* [19], as well as bacteria of the genera *Rickettsia* [20], *Arsenophonus* and *Wolbachia* [21].

There are reports about the detection of viruses in *M. ovinus*, such as Bluetongue virus and Border disease virus, which cause sheep and goat diseases [22,23]. *Melophagus ovinus* was shown to be able to support the replication of the dengue virus [24]. The metagenomic approach has allowed the discovery of several new viruses in various species of louse flies. Seven new viruses were discovered in bat flies (Hippoboscoidea: Nycteribiidae and Streblidae) from Mexico and Uganda [25]. Using a similar approach, ten new viruses from several divergent viral groups were detected in the pools of unidentified Hippoboscidae from Hubei, China [1]. However, as of yet, there have been no studies revealing the virome of *M. ovinus* using modern metagenomic approaches.

In this work, we report the findings of our study into the virome of *M. ovinus*, which was collected in the Republic of Tuva, Russia. We were able to identify five novel viruses and test their ability to replicate in the pig embryo kidney (PEK) cell line.

## 2. Materials and Methods

### 2.1. Collection and Pooling of Melophagus ovinus

Sheep ked specimens were collected from domestic sheep in 2010 and 2012 in the Republic of Tuva, Russia (see Table 1). The exact species of the louse flies were confirmed using contigs containing mitochondrial DNA after next generation sequencing was performed.

### 2.2. Sample Preparation and High-Throughtput Sequencing

Individual specimens of *M. ovinus* were homogenized using Tissue Lyser 2 (12 min, frequency 25 s^−1^). Prior to extraction of the nucleic acid, aliquots of the homogenate of keds collected from the same animal were pooled together in equal amounts. RNA was extracted from pooled homogenates using TRI Reagent LS (Sigma, St. Louis, MA, USA) according to the manufacturer’s instructions. After extraction, host rRNA was depleted using a NEBNext Globin and rRNA Depletion Kit (NEB, E7750S, Ipswich, MA, USA) according to the manufacturer’s instructions. The obtained RNA was used for library preparation without polyA-enrichment using a NEBNext Ultra II RNA Library Prep Kit for Illumina (NEB, E7770, Ipswich, MA, USA) according to the manufacturer’s instructions. Final libraries were sequenced (single-end, 250-nt reads) on a HiSeq1500 (Illumina, San Diego, CA, USA). Raw reads were deposited in the sequence read archive (BioProject accession number PRJNA777535).

### 2.3. Sanger Sequencing

For Sanger sequencing, RNA was extracted from homogenates of the individual specimens using TRI Reagent LS (Sigma, St. Louis, MO, USA) according to the manufacturer’s instructions. Reverse transcription was carried out using an MMLV RT kit (Evrogen, Moscow, Russia) with a random hexamer primer according to the manufacturer’s instructions. cDNA was used for a PCR with DreamTaq DNA polymerase (Thermo Fisher Scientific, Vilnius, Lithuania) using virus-specific oligonucleotides (Table S1). PCR fragments were gel-purified with a QIAquick Gel Extraction Kit (QIAGEN, Hilden, Germany) and then sequenced with an Applied Biosystems 3500 genetic analyzer (Waltham, MA, USA) using a BigDye Terminator v3.1 Cycle Sequencing Kit (Thermo Fisher Scientific, Vilnius, Lithuania). The Obtained sequences were aligned in SeqMan v.7.0.0 using contigs from high-throughput sequencing as reference sequences.

### 2.4. Assembly and Analysis

Adapter sequences, bases with low quality (<Q30) and short reads (length < 35) were discarded using Trimmomatic v0.39 [26]. Trimmed reads were used for de novo contig assembly with SPAdes v3.13.0 [27]. The resultant contigs were screened for viral sequences using the blastn algorithm in BLAST v2.9.0+ with the nt database, and contigs containing virus-related sequences were extracted for further investigation. Open reading frames were extracted from such contigs and tested using the blastp algorithm to determine whether they were virus related.

For all contigs that showed a relation to viruses of the same family, an estimation of evolutionary divergence was performed to estimate whether all of them belonged to the same virus species. All contigs were aligned in the Mega X program. The obtained alignment then was used as the input datum to compute pairwise distances in the Mega X program with the default settings [28].

Contigs containing a complete coding region for the virus were extracted and used for further studies. In cases when there were several very closely related contigs in the same *M. ovinus* pool, Sanger sequencing of individual ked suspensions was performed. Contigs with the closest identities to the fragments from Sanger sequencing were used as viral sequences for further studies. In some cases, we were unable to perform a PCR due to a lack of material, or the PCR was negative. In that case, the consensus sequences were recomputed using the longest contig sequence as a reference with uGene v.1.32.0 [29] (up to 10% mismatches allowed). Obtained virus sequences were deposited in the GenBank database (accession numbers OL420682-OL420732).

Prevalence of the viral reads in each pool was estimated by aligning reads from the pool on the sequences of viral contigs using Bowtie2 v.2.3.5.1 software [30]. The reported percentage of the reads uniquely aligned to the viral genome was considered as a percentage of the viral reads in the probe.

### 2.5. Phylogenetics and Visualization

From the obtained contigs, either the polyprotein (if available) or RNA-dependent polymerase protein sequence was extracted. This sequence, along with homologs, was aligned using MAFFT v7.310 [31]. Alignments were processed with the TrimAL v1.4. rev 15 [32] program to remove ambiguously aligned regions, and maximum-likelihood phylogenetic trees were constructed with the phyML 3.3.20200621 [33] program with 1008 bootstrap replications. Phylogenetic trees were visualized in FigTree v.1.4.4.

Genomes of the viruses were drawn using custom Python script. All post-processing of the images was performed with the GIMP v.2.10.24 program.

### 2.6. Virus Passages in Pig Embryo Kidney Cell Line

A PEK cell line was used to assess the ability of the identified viruses to replicate in mammalian cells. The PEK cell line was maintained at 37 °C in Medium 199, Earle’s Salts (FSASI Chumakov FSC R&D IBP RAS, Moscow, Russia), supplemented with 5% fetal bovine serum (FBS, Gibco, Paisley, UK).

For the experiment, cells were seeded in flat-sided cell culture tubes (NUNC, Thermo Fisher Scientific, Roskilde, Denmark) and cultivated for one to two days for a final cell count of 0.5–2 × 10^−6^ cells per tube. Then, cells were infected, either with 200 μL of the homogenate of keds or with 200 μL of the cultural fluid collected from the previous virus passage, and incubated in the thermostat at 37 °C. After four days, the culture supernate was collected and stored.

## 3. Results

### 3.1. High-Throughput Sequencing and Detection of Virus-like Contigs

We processed five pools of the sheep ked *M. ovinus* (two to six specimens in each pool) collected in the Republic of Tuva, Russia, in 2010 and 2012. No previously known viruses were identified in the samples, but five distinct types of contigs with homology to the viral polymerases were identified. All of them were close to the various groups of the RNA viruses, *Rhabdoviridae* (*Sigmavirus*), *Iflaviridae*, *Reoviridae* and *Solemoviridae*.

### 3.2. Iflaviridae—Related Contigs

Classical iflaviruses are non-enveloped, single-stranded, non-segmented, positive-sense RNA viruses. The genome has a 9–11 kb length and encodes a single open reading frame (ORF). This is translated into the single polyprotein that is processed into structural (N-terminus) and non-structural proteins. All the iflaviruses were isolated from arthropods [34].

**Figure 1:**
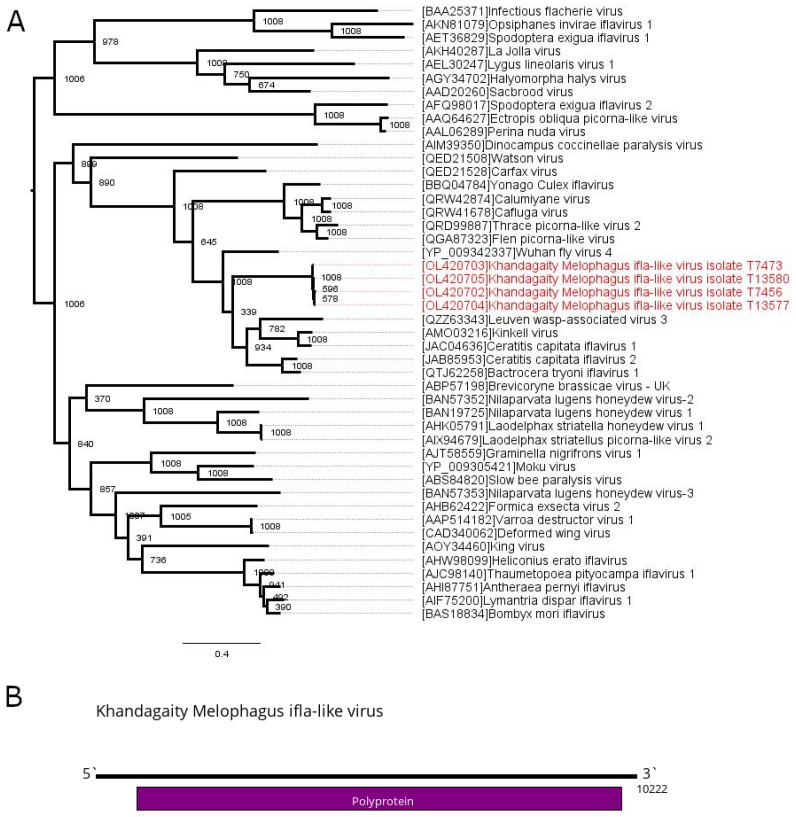
Phylogenetic relationships and genomic structure of the Khandagaity Melophagus ifla-like virus (KMIV). (**A**) Phylogenetic relationships of the KMIV. Analysis was performed using amino acid sequences of the polyprotein, with 1008 bootstrap replicates. Bootstrap support is shown at each node. Scale bar represents the number of amino acid substitutions per site. Viruses discovered in the current article are marked in red. Tree is midpoint rooted for the clarity of the figure only. (**B**) Scheme of the KMIV genome.

In the current work, we found contigs exhibiting similarities with members of *Iflaviridae* in all of the studied pools. Contigs from all the pools except pool #21 had the typical length for iflaviruses (around 10,200 nt) and encoded a single ORF ~3050 amino acids in length (Figure 1). They were not identical, with nucleotide divergence from 0.0002 to 0.0984 for different sequence pairs (Table S2). A protein blast of the polyprotein sequence showed around 43% identity with 96% coverage to the closest relative (Bactrocera tryoni iflavirus 1). The data showed that all of these contigs belong to the single novel virus named Khandagaity Melophagus ifla-like virus (KMIV). We then reassembled full virus genomes (Section 2.4) and obtained four full-genome sequences.

KMIV reads were rare, accounting for 0.02–0.14% of the total reads in the pools (Table 2). The low abundance (0.02%) of the viral reads in pool #21 was likely the reason we were not able to assemble the full genome from its data.

According to phylogenetic analysis of the polyprotein sequence, KMIV forms a monophyletic group with, related to the family *Iflaviridae* but yet unclassified, Ceratitis capitata iflavirus 1 and Ceratitis capitata iflavirus 2, which were found in the Mediterranean fruit fly (*Ceratitis capitata*) [34,35]. Other close relatives of KMIV are fly iflaviruses, such as Wuhan fly virus 4 [2], Kinkell virus [36], Bactrocera tryoni iflavirus 1 [37] and the Leuven wasp-associated virus 3 identified in the common wasp (*Vespula vulgaris*) [38] (Figure 1).

### 3.3. Solemoviridae—Related Contigs

Classical solemoviruses are non-enveloped viruses with an ~4.5 kb positive sense RNA genome that infects different groups of flowering plants. They rely on −1 ribosomal frameshifting, leaky scanning and generating subgenomic RNA to produce their proteins. The RNA-dependent RNA polymerase (RdRp) of *Solemoviridae* is phylogenetically close to the RdRps of the *Luteoviridae* family. Recently, many new viruses with RdRps related to *Solemoviridae* and *Luteoviridae* were discovered. Some of those novel viruses differed drastically in their overall genome structure, for example, by having a different ORF count and/or by splitting the genome into two segments [2].

Here, we discovered multiple solemoviridae-related contigs in all of the studied pools. It should be noted, however, that said contigs were not closely related to the classical solemoviruses, but instead were closely related to the novel segmented solemoviridae-like viruses. The contigs from our data formed two separate clusters, with little homology between the clusters. Even within each cluster, the contigs were relatively diverse (up to a 0.22-nucleotide divergence) (Table S5). A protein blast of the putative polymerase showed ~43% identity to the closest relative (Teise virus) for the first cluster of contigs. Hubei diptera virus 14 was the closest to the contigs in the second cluster with 59% identity in the polymerase. Using the second segment of closest viruses, we were able to recover the second segment for each cluster (Table S6). A protein blast of the first cluster of contigs (the first ORF on the second segment) showed 41% identity to the Motts Mill virus (second closest, Teise virus). The second segment for the second cluster of sequences showed 47–48% identity to the Hubei solemo-like virus based on the amino acid sequences of the first ORF. These data show that these two clusters of contigs belong to two separate novel viruses. The contigs from the first cluster were reassembled (Section 2.4) and named Bayan-Khairhan-Ula Melophagus solemo-like virus (BKUMSV), while the contigs from the second were named Ulaatai Melophagus solemo-like virus (UMSV).

**Figure 2:**
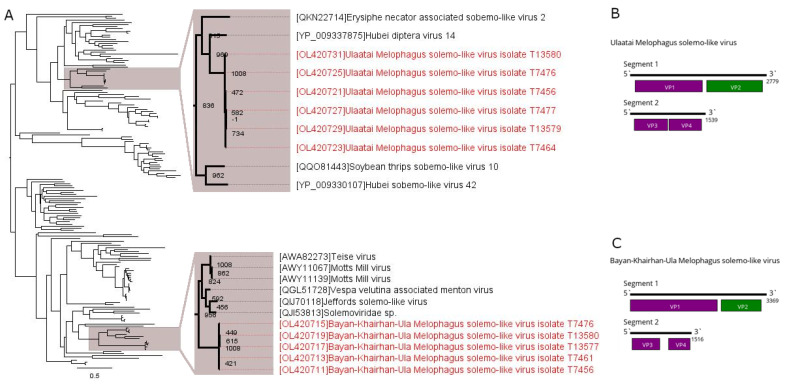
Phylogenetic relationships and genomic structure of the Ulaatai (UMSV) and Bayan-Khairhan-Ula Melophagus solemo-like viruses (BKUMSV). (**A**) Phylogenetic relationships within solemo-like viruses. Analysis was performed using amino acid sequences of the polymerase, with 1008 bootstrap replicates. Bootstrap support is shown at each node. Scale bar represents the number of amino acid substitutions per site. Viruses discovered in the current article are marked in red. Tree is midpoint rooted for the clarity of the figure only. (**B**) Scheme of the UMSV genome. (**C**) Scheme of the BKUMSV genome. RNA-dependent RNA polymerase is marked in green.

UMSV and BKUMSV were found in all of the pools. The abundance of these viruses was close and varied between 0.41 and 1.17% (Table 2). These viruses belong to different parts of the Luteo-Solemo supergroup, but they have similar genome structures (Figure 2). They contain two segments, with four ORFs located on them. The first segment is large: ~3400 nt in BKUMSV and ~2800 nt in UMSV. It encodes putative peptidase and polymerase. The second segment is smaller (~1500 nt for both viruses). The ORFs on the second segment likely take part in viral coat protein production. These two frames are divided by a single UAG stop-codon, making it likely that the mechanism of expression involves a stop-codon read-through.

Phylogenetic analysis based on the polymerase sequence showed that UMSV formed a monophyletic group with Hubei diptera virus 14 (found in the pool of diptera) [2] and Erysiphe necator-associated solemo-like virus 2 (discovered in the *Erysiphe necator* fungus) (Figure 2A). The other closely related viruses are Hubei solemo-like virus 42 [2] and Soybean thrips solemo-like virus 10 discovered in arthropods [39].

BKUMSV forms a monophyletic group with Teise, Prestney Burn, Motts-Mill (detected in *Drosophila* species) [40,41], Jeffords solemo-like virus (*Musca vetustissima* flies) [42], Vespa velutina-associated menton virus (detected in *Vespa velutina* Asian hornets) [43] and a novel virus found in the metagenome of the brown shrike (*Lanius cristatus*) anal swab (Figure 2A).

### 3.4. Sigmavirus—Related Contigs

Sigmaviruses (*Rhabdoviridae*) are common pathogens of Drosophilidae, and recently, they were found in the other members of Diptera and other arthropods. They have a negative-sense RNA genome of around ~12.5 kb in length, encoding five to six genes. In addition to the five proteins classical for rhabdoviruses (L, G, M, P, N), they may have an additional protein X encoded between the P and M genes, or another additional ORF between the G and L genes [44].

**Figure 3:**
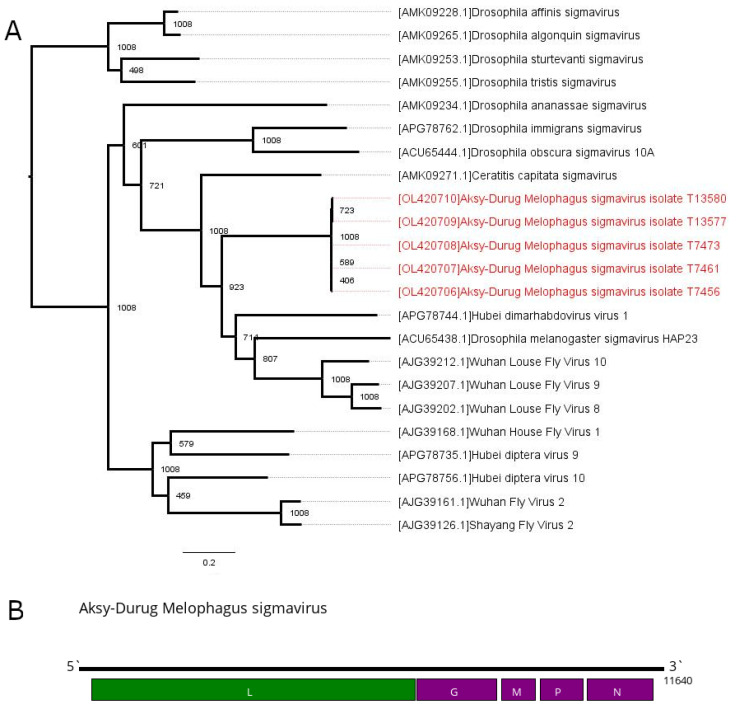
Phylogenetic relationships and genomic structure of the Aksy-Durug Melophagus sigmavirus (ADMSV). (**A**) Phylogenetic relationships within genus *Sigmavirus*. Analysis was performed using amino acid sequences of the polymerase, with 1008 bootstrap replicates. Bootstrap support is shown at each node. Scale bar represents the number of amino acid substitutions per site. Viruses discovered in the current article are marked in red. Tree is midpoint rooted for the clarity of the figure only. (**B**) Scheme of the ADMSV genome. RNA-dependent RNA polymerase is marked in green.

In the current work, we found contigs exhibiting similarities with members of genus *Sigmavirus* in all of the studied pools. All the contigs had a classical *Rhabdoviridae* genome structure, encoding the L, G, M, P and N genes (Figure 3), but not encoding protein X. Most of the contigs were ~11.6 kb in length; however, one from pool #22 was shorter (10,988 nt) and had a shortened polymerase sequence. Apart from this, the recovered contigs have low divergence in comparison to each other (from 0.00119 to 0.05223) (Table S3).

In the genus *Sigmavirus*, there are species discrimination criteria established by the International Committee on the Taxonomy of Viruses. These contigs match both criteria for the new species. They have low identity in RdRp to the closest relative (around 53% to the Wuhan Louse Fly Virus 9) and occupy a different ecological niche, as there is no other sigmavirus that infects *M. ovinus*. Taking these data into account, we conclude that all of these contigs belong to the single novel virus named Aksy-Durug Melophagus sigmavirus (ADMSV). ADMSV sequence abundances were relatively high in pools #21–24, representing 1.97–4.76% of the total reads, while being low (0.15%) in pool #20 (Table 2).

Phylogenetic analysis based on the sequence of the L gene places ADMSV within the *Sigmavirus* genus. ADMSV forms a well-defined group that includes several viruses of louse flies (Wuhan louse fly viruses 8, 9 and 10) [1], fruit flies (Drosophila melanogaster sigmavirus HAP23 and Ceratitis capitata sigmavirus) [44] and unidentified diptera (Hubei dimarhabdovirus virus 1) [2] (Figure 3A).

### 3.5. Reoviridae—Related Contigs

Viruses of the family *Reoviridae* have double-stranded linear RNA genomes, separated in the 9, 10, 11 or 12 segments. Viral RNAs are mostly monocistronic, although in some cases, a second ORF is present. Proteins are always encoded on only one strand of the RNA duplex. The biological properties of reoviruses are quite diverse. Some viruses infect only vertebrates or invertebrates, some are known arboviruses and some viruses replicate in both plants and arthropod vectors [45].

**Figure 4:**
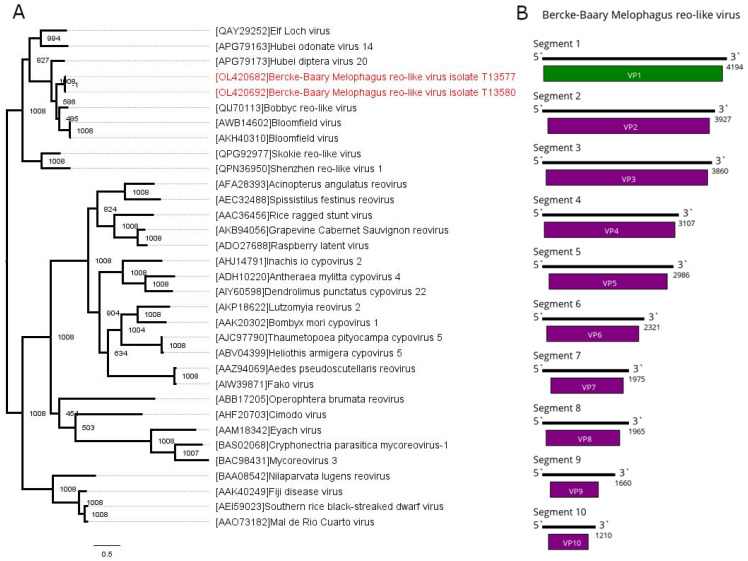
Phylogenetic relationships and genomic structure of the Bercke-Baary Melophagus reo-like virus (BBMRV). (**A**) Phylogenetic relationships of the BBMRV. Analysis was performed using amino acid sequences of the polymerase, with 1008 bootstrap replicates. Bootstrap support is shown at each node. Scale bar represents the number of amino acid substitutions per site. Viruses discovered in the current article are marked in red. Tree is midpoint rooted for the clarity of the figure only. (**B**) Scheme of the BBMRV genome. RNA-dependent RNA polymerase is marked in green.

In the current work, we found contigs with similarities to the polymerases (first segment) of the reoviridae-like viruses in pools #23 and 24. A subsequent search allowed us to identify nine more contigs from each pool with homologies to the second to 10th segments of the reoviridae-like viruses. The segments found in the different pools were very close to each other (0.0005–0.029 nucleotide difference). At the same time, they were considerably different from any entries found in the GenBank (28–61% identity, dependent on the segment) (Table S4). This allowed us to conclude that these 10 contigs from each pool represent a single novel reoviridae-like virus. It was named Bercke-Baary Melophagus reo-like virus (BBMRV). The complete BBMRV genome consists of 10 genome segments, with the first one, which contains putative RdRp, being the largest (~4000 nt) and the tenth segment being the smallest (~1200 nt) (Figure 4B). Each segment encodes one ORF. Based on the nucleotide sequence of segment one, the BBMRVs found in pools 23 and 24 were extremely similar, with only a 0.0005 divergence.

In the pools where BBMRV was found, its presence was higher than the abundance of any other virus found in the current work: 19.36% in pool 23 and 54.33% in pool 24 (Table 2). Phylogenetic analysis of the sequence of the viral polymerase places BBMRV together with various reoviruses of Diptera, such as Hubei diptera virus 20 [2], Bobbyc reo-like virus [42] and Bloomfield virus [40] (Figure 4A).

### 3.6. Multiplication of Viruses in Mammalian Cells

Here, we studied the virome of the obligate blood-sucking ectoparasite *M. ovinus*. Such a lifestyle implies that the viruses it harbors may be transmitted to the mammalian host and potentially cause illness. We tested the ability of the discovered viruses to infect mammalian cells. We used a PEK cell as a model culture because it was previously shown to be able to support the reproduction of different arboviruses, including orbiviruses [46].

We infected a PEK cell with suspensions of the individual keds and then performed a second passage by infecting fresh PEK cells with cultural supernate from infected PEK cells. After this, we tested the supernate collected from the first, second and third passages for the presence of all five viruses described here using virus-specific oligonucleotides (Table S1). Each PCR-positive result was confirmed using Sanger sequencing of the obtained PCR product. When a PCR-positive result was not confirmed using Sanger sequencing, the probe was considered negative.

BBMRV was detected in ked suspensions #13578 and 13579, and after the first passage of suspensions #13578 and 13580. BKUMSV was detected in ked suspensions #7476 and 7477 and the first passage of suspension #13580. UMSV was detected in ked suspensions #7458, 7459, 7460, 7464, 7476, 7477, 13578 and 13579 and the first passage of suspension #13580 (Table 3).

KMIV was detected in ked suspensions #7457 and 13578, in the first passage of suspension #13581, in suspension #7456 only at the second passage, in suspension 13580 in both the first and second passages and in suspension #13579 and in the PEK cell supernate after second passage. ADMSV was detected in suspension #7464, in suspension #13581 only in the first passage, in suspension #7473 in the second passage and in suspensions #13579 and 7477 in the PEK cell supernate after the first and second passages. Moreover, ADMSV was detected in the suspension #7474 line throughout the three passages. Thus, ADMSV was detected in the first, second and third passages of the ked suspensions in the PEK cell culture (Figures S1–S4). This may indicate the ability of this virus to replicate in mammalian cells.

## 4. Discussion

In the last decade, meta-transcriptomic studies have revealed the extensive diversity of the RNA viruses of invertebrates [1,2]. Some of the new viruses are considered to be pathogenic arboviruses and are being extensively studied [46,47,48,49,50]. While the viromes of some blood-sucking ectoparasites, such as mosquitoes and ticks, are relatively well-studied [6,7,9,10], there are few data on less-known species, such as louse flies [2]. Here, we present data on the virome of *M. ovinus*, a widely distributed sheep ectoparasite. Five pools of two to six specimens collected in the Republic of Tuva, Russia, were studied.

Bluetongue virus and Border disease virus were previously detected in the sheep ked [22,23]; however, no known pathogenic viruses were found in the present study. We were able to assemble five full genomes of novel viruses. All of the novel viruses were fairly divergent from known viruses. They belonged to the four major virus groups—*Iflaviridae*, *Rhabdoviridae*, *Reoviridae Solemoviridae*—representing different genome coding strategies. KMIV and ADMSV had a genome structure similar to the well-known viruses within their supposed virus family.

A segmented structure of some solemoviridae-like viruses was discovered recently, with the genome divided into two separate segments [2]. While we were able to recover both segments from our data, some of the closely related viruses—for example, Erysiphe necator associated solemo-like virus 2, Prestney Burn virus and Jeffords solemo-like virus—only have a sequence homological to segment one in the databases (as of 26 October 2021). This is even more relevant for reoviridae-like viruses. There are nine known segments of the Bloomfield virus, three for Hubei odonate virus 14, two for Hubei diptera virus 20 and only polymerase-encoding for Bobbyc reo-like virus and the Elf-Loch viruses [2,40,42]. Such a situation can arise for various reasons, ranging from a low prevalence of the virus in the sample to the loose homology of some proteins, making them hard to identify as viral proteins with standard procedures. In the current work, many of the proteins of BBMRV had significant similarity via a protein blast only to the homolog proteins of the Bloomfield virus. Moreover, we cannot even be certain that BBMRV has 10 segments, and not 12, as with some of the *Reoviridae*, as they may be left unidentified due to the low homology. The segment count not only remains one of the taxon-defining features of the *Reoviridae* [45] but can also shed light on the evolution of these viruses. Thus, it is crucial to try to recover as many viral segments as possible from the data obtained.

*Melophagus ovinus* is a blood-sucking insect, and it was collected directly from sheep in our study. This means it is possible that the viruses discovered in this work can infect *M. ovinus*, sheep or circulate between sheep and keds (as arboviruses). The majority of the closest relatives of the discovered viruses are various viruses found in other species (mostly non-parasitic) of the Diptera order. Such phylogenetic relationships imply that all the discovered viruses are arthropod-specific; however, we cannot exclude the possibility of them being sheep viruses (with viral RNA detected in the blood carried by *M. ovinus*) or arboviruses. Indeed, the viruses discovered in the current work only have a loose homology to the previously known viruses, and there are examples of the purely insect-specific viruses being closely related to arboviruses and vertebrate viruses [51]. We decided to test the ability of the discovered viruses to replicate in the mammalian cells in the PEK cell model. Three passages were performed. All the viruses were detected in at least one line after the first passage, while two (KMIV and ADMSV) were detected after both the first and second passages. Only ADMSV was detected after the third passage.

While detection after the first passage could be explained by detecting diluted RNA from the original ked suspension, it is less likely to be the case for the second passage and even less for the third. With ADMSV being continuously detected throughout three passages, we suggest that it is able to replicate in the mammalian PEK cells.

However, we were not able to detect KMIV after the third passage in the PEK cells. It suggests that we were detecting a diluted virus from the ked suspension during the first and second passages. At the same time, in some cases, we were able to detect KMIV in the second passage while not detecting it on the first one (Table 3). Thus, it is possible that KMIV might replicate in the PEK cells on the low level, with our primer pair not being sensitive enough to always detect it. Additional experiments are needed to come to a conclusion on the ability of KMIV to replicate in the PEK cells.

According to the definition of the Subcommittee on the Evaluation of Arthropod-Borne Status, all the viruses can be divided as follows: (1) arbovirus, (2) probable arbovirus, (3) possible arbovirus, (4) probably not arbovirus, (5) not arbovirus. Categories one and five include viruses with their status proven beyond reasonable doubt. If the data on the virus arbovirus nature fail to meet strong criteria, such viruses are registered in categories two and four. The viruses included in category three (possible arbovirus) have data too meager for firm judgment [52]. The phylogenetic relationships of ADMSV suggest that it might be an insect virus. At the same time, our data also imply that it may be able to replicate in mammalian PEK cells. Such data hit on the possibility of ADMSV being an arbovirus, but this is not enough to conclude the arbovirus nature of this virus. This would mark ADMSV as a possible arbovirus, as per the abovementioned definition. Additional data on both replication in the mammalian cell cultures and evidence on vector–host cycling are needed to determine its arbovirus nature.

There are known *Rhabdoviridae* arboviruses [5]. However, sigmaviruses are known to only be transmitted vertically [53]. Recently, it has been speculated that some of the viruses of louse flies may infect bats [54]. Our data suggest that ADMSV—a louse fly derived sigmavirus—may be able to replicate in mammalian cells. Overall, it seems that the routes of transmission of blood-sucking dipteran sigmaviruses should be studied more thoroughly.

## 5. Conclusions

We studied the virome of *M. ovinus*, a blood-sucking ectoparasite of sheep. The full genomes of five novel viruses were assembled. Phylogenetically, all the discovered viruses clustered mostly with dipteran viruses. Our data suggest that Aksy-Durug Melophagus sigmavirus may be able to replicate in mammalian cells. Such data mark this virus as a possible arbovirus, and additional research is needed to precisely assess its biology.

## Figures and Tables

**Table 1:** Collection of *Melophagus ovinus* specimens.

Pool Number	Sheep Number	Specimen Number in the Pool	Location	Collection Date
20	1	5	50.73376, 92.26536	2010
21	1	6	50.73376, 92.26536	2010
22	2	5	50.73376, 92.26536	2010
23	3	3	50.73727, 92.26536	2012
24	3	2	50.73727, 92.26536	2012

**Table 2:** Presence of virus-containing reads in each pool of *M. ovinus*.

Pool Number	Number of Reads after Filtering	KMIV ^1^	UMSV ^2^	BKUMSV ^3^	ADMSV ^4^	BBMRV ^5^	Virus Reads Total
20	7 852 210	0.14%	1.17%	1.03%	0.15%		2.49%
21	8 033 216	0.02%	0.77%	0.41%	2.42%		3.62%
22	6 958 480	0.07%	0.63%	0.43%	1.97%		3.10%
23	7 527 561	0.09%	0.97%	0.46%	4.76%	19.36%	25.64%
24	4 622 179	0.12%	0.47%	1.07%	2.35%	54.33%	58.34%

^1^ Khandagaity Melophagus ifla-like virus. ^2^ Ulaatai Melophagus solemo-like virus. ^3^ Bayan-Khairhan-Ula Melophagus solemo-like virus. ^4^ Aksy-Durug Melophagus sigmavirus. ^5^ Bercke-Baary Melophagus reo-like virus.

**Table 3:** Detection of novel viruses during passages in PEK cells.

Ked Suspension		Pool Number	Viruses Detected in the 1st Passage	Viruses Detected in the 2nd Passage	Viruses Detected in the 3rd Passage ^2^
Number	Viruses Detected ^1^				
7456	no	20	no	KMIV	no
7457	KMIV		no	no	nd
7458	UMSV		no	no	nd
7459	UMSV		no	no	nd
7460	UMSV		no	no	nd
7461	no	21	no	no	nd
7462	no		no	no	nd
7463	no		no	no	nd
7464	ADMSV/UMSV		no	no	nd
7465	no		no	no	nd
7466	nd		no	no	nd
7473	no	22	no	ADMSV	no
7474	ADMSV		ADMSV	ADMSV	ADMSV
7475	no		no	no	nd
7476	UMSV/BKUMSV		no	no	nd
7477	ADMSV/UMSV/BKUMSV		ADMSV	ADMSV	no
13577	no	23	no	no	nd
13578	BBMRV/ADMSV/KMIV/UMSV		BBMRV/ADMSV	no	no
13579	BBMRV/ADMSV/KMIV/UMSV		ADMSV	ADMSV/KMIV	no
13580	nd	24	all	KMIV	nd
13581	no		ADMSV/KMIV	no	nd

^1^ Virus RNA detected with virus-specific oligonucleotides. **no**—no virus detected. **ADMSV**—Aksy-Durug Melophagus sigmavirus, **BBMRV**—Bercke-Baary Melophagus reo-like virus, **KMIV**—Khandagaity Melophagus ifla-like virus, **UMSV**—Ulaatai Melophagus solemo-like virus, **BKUMSV**—Bayan-Khairhan-Ula Melophagus solemo-like virus, **all**—all viruses discovered in this article were detected, **nd**—no data. ^2^ At the third passage cultural supernate was tested only for KMIV and ADMSV.

## Data Availability

Raw high throughput sequencing data obtained during this study are available in the SRA database (BioProject accession number PRJNA777535). Obtained virus sequences were deposited in the GenBank database (accession numbers OL420682-OL420732).

## Supplementary Materials

The following are available online at https://www.mdpi.com/article/10.3390/v13122375/s1, Table S1: Oligonucleotides used for virus detection, Table S2: Estimates of evolutionary divergence between Ifla-like contigs found in the study, Table S3: Estimates of evolutionary divergence between sigmavirus-like contigs found in the study, Table S4: Estimates of evolutionary divergence between reo-like contigs and closes relative for each segment found in the GenBank database, Table S5: Estimates of evolutionary divergence between solemoviridae-like contigs (first segment) found in the study, Table S6: Estimates of evolutionary divergence between solemoviridae-like contigs (second segment) found in the study, Figure S1: Electrophoresis of ADMSV PCR-detection in the ked suspensions, Figure S2: Electrophoresis of ADMSV PCR-detection after the first passage in the PEK cells, Figure S3: Electrophoresis of ADMSV PCR-detection after the second passage in the PEK cells, Figure S4: Electrophoresis of ADMSV PCR-detection after the third passage in the PEK cells.

## References

[1] Li C., Shi M., Tian J., Lin X., Kang Y., Chen L., Qin X., Xu J., Holmes E.C., Zhang Y. (2015). Unprecedented genomic diversity of RNA viruses in arthropods reveals the ancestry of negative-sense RNA viruses. Elife.

[2] Shi M., Lin X., Tian J., Chen L., Chen X., Li C., Qin X., Li J., Cao J., Eden J. (2016). Redefining the invertebrate RNA virosphere. Nature.

[3] Paraskevopoulou S., Ka S., Zirkel F., Donath A., Petersen M., Liu S., Zhou X., Drosten C., Misof B., Junglen S. (2021). Viromics of extant insect orders unveil the evolution of the flavi-like superfamily. Virus.

[4] Dolja V.V., Koonin E.V. (2018). Metagenomics reshapes the concepts of RNA virus evolution by revealing extensive horizontal virus transfer. Virus Res..

[5] Young P.R., Hilgenfield R., Vasudevan S. (2018). Arboviruses: A Family on the Move. Dengue and Zika: Control and Antiviral Treatment Strategies.

[6] Marseille R., Nebbak A., Monteil-bouchard S., Berenger J., Almeras L., Parola P., Desnues C. (2021). Virome Diversity among Mosquito Populations in a Sub-Urban Region of Marseille, France. Viruses.

[7] Atoni E., Wang Y., Karungu S., Waruhiu C., Zohaib A., Obanda V., Agwanda B., Mutua M., Xia H., Yuan Z. (2018). Metagenomic Virome Analysis of Culex Mosquitoes from Kenya and China. Viruses.

[8] Hameed M., Wahaab A., Shan T., Wang X., Khan S., Di D., Xiqian L., Zhang J.-J., Anwar M.N., Nawaz M. (2021). A Metagenomic Analysis of Mosquito Virome Collected From Different Animal Farms at Yunnan—Myanmar Border of China. Front. Microbiol..

[9] Harvey E., Rose K., Eden J., Lo N., Abeyasuriya T., Shi M., Doggett S.L., Holmes E.C. (2019). Extensive Diversity of RNA Viruses in Australian Ticks. J. Virol..

[10] Pettersson J.H., Shi M., Bohlin J., Eldhol V., Brynildsrud O.B., Paulsen K.M., Andreassen Å., Holmes E.C. (2017). Characterizing the virome of Ixodes ricinus ticks from northern Europe. Sci. Rep..

[11] Tetley J.H. (1958). The sheep ked, Melophagus ovinus L. I. Dissemination potential. Parasitology.

[12] Karbowiak G., Demiaszkiewicz A.W., Pyziel A.M., Wita I., Moskwa B., Werszko J., Bień J., Goździk K., Lachowicz J., Cabaj W. (2014). The parasitic fauna of the European bison (Bison bonasus) (Linnaeus, 1758) and their impact on the conservation. Part 1 The summarising list of parasites noted. Acta Parasitol..

[13] Sertse T., Wossene A. (2007). Effect of ectoparasites on quality of pickled skins and their impact on the tanning industries in Amhara regional state, Ethiopia. Small Rumin. Res..

[14] Jiang B.-G., Qiu E.-C., Zhang F., Zuo S.-Q., Yang H., Liu W., Cao W.-C. (2011). Borrelia burgdorferi sensu lato in sheep keds (Melophagus ovinus), Tibet, China. Vet. Microbiol..

[15] Zhang Q., Wang Y., Li Y., Han S., Wang B., Yuan G., Zhang P., Yang Z., Wang S., Chen J. (2021). Vector-Borne Pathogens with Veterinary and Public Health Significance in Melophagus ovinus (Sheep Ked) from the Qinghai-Tibet Plateau. Pathogens.

[16] Zhao L., He B., Li K., Li F., Zhang L., Li X., Liu Y. (2018). First report of Anaplasma ovis in pupal and adult Melophagus ovinus (sheep ked) collected in South Xinjiang, China. Parasit. Vectors.

[17] Liu Y., He B., Li F., Li K., Zhang L., Li X., Zhao L. (2018). Molecular Identification of Bartonella melophagi and Wolbachia Supergroup F from Sheep Keds in Xinjiang, China. Korean J. Parasitol..

[18] Hao L., Yuan D., Li S., Jia T., Guo L., Hou W., Lu Z., Mo X., Yin J., Yang A. (2020). Detection of Theileria spp. in ticks, sheep keds (Melophagus ovinus), and livestock in the eastern Tibetan Plateau, China. Parasitol. Res..

[19] Martinkovic F., Matanovic K., Rodrigues A.C., Garcia H.A., Teixeira M.M.G. (2012). Trypanosoma (Megatrypanum) melophagium in the Sheep Ked Melophagus ovinus from Organic Farms in Croatia: Phylogenetic Inferences Support Restriction to Sheep and Sheep Keds and Close Relationship with Trypanosomes from Other Ruminant Species. J. Eukaryot. Microbiol..

[20] Liu D., Wang Y., Zhang H., Liu Z., Wureli H., Wang S., Tu C., Chen C. (2016). First report of Rickettsia raoultii and R. slovaca in Melophagus ovinus, the sheep ked. Parasit. Vectors.

[21] Duan D.Y., Zhou H.M., Cheng T.Y. (2019). Comparative analysis of microbial community in the whole body and midgut from fully engorged and unfed female adult Melophagus ovinus. Vet. Entomol..

[22] Liu Y.-H., He B., Li K., Li F., Zhang L., Li X., Zhao L. (2019). First report of border disease virus in Melophagus ovinus (sheep ked) collected in Xinjiang, China. PLoS ONE.

[23] Luedke A.J., Jochim M.M., Bowne J.G. (1965). Preliminary Bluetongue Transmission with the Sheep Ked Melophagus Ovinus. Can. J. Comp. Med. Vet. Sci..

[24] Setién Á.A., Baltazar A.G., Leyva I.O., Rojas M.S., Koldenkova V.P., Garcia M.P.-P., Ceballos N.A., Romero G.G., Villegas E.O.L., Malacara J.B.M. (2017). Ectoparasitic hematophagous dipters: Potential reservoirs of dengue virus?. Gac. Med. Mex..

[25] Ramirez-Martinez M., Bennett A.J., Dunn C.D., Yuill T.M., Goldberg T.L. (2021). Bat Flies of the Family Streblidae (Diptera: Hippoboscoidea) Host Relatives of Medically and Agriculturally Important. Viruses.

[26] Bolger A.M., Lohse M., Usadel B. (2014). Trimmomatic: A flexible trimmer for Illumina sequence data. Bioinformatics.

[27] Bankevich A., Nurk S., Antipov D., Gurevich A.A., Dvorkin M., Kulikov A.S., Lesin V.M., Nikolenko S.I., Pham S., Prjibelski A.D. (2012). SPAdes: A new genome assembly algorithm and its applications to single-cell sequencing. J. Comput. Biol..

[28] Kumar S., Stecher G., Li M., Knyaz C., Tamura K. (2018). MEGA X: Molecular Evolutionary Genetics Analysis across Computing Platforms. Mol. Biol. Evol..

[29] Okonechnikov K., Golosova O., Fursov M., The UGENE Team (2012). Unipro UGENE: A unified bioinformatics toolkit. Bioinformatics.

[30] Langmead B., Salzberg S. (2013). Fast gapped-read alignment with Bowtie 2. Nat. Methods.

[31] Kazutaka K., Standley D.M. (2013). Standley MAFFT multiple sequence alignment software version 7: Improvements in performance and usability. Mol. Biol. Evol..

[32] Capella-Gutierrez S., Silla-Martinez J.M., Gabaldon T. (2009). trimAl: A tool for automated alignment trimming in large-scale phylogenetic analyses. Bioinformatics.

[33] Guindon S., Gascuel O. (2003). PhyML: “A simple, fast, and accurate algorithm to estimate large phylogenies by maximum likelihood”. Syst. Biol..

[34] Valles S.M., Chen Y., Firth A.E., Gu D.M.A., Hashimoto Y., Herrero S., De Miranda J.R., Ryabov E. (2017). ICTV Virus Taxonomy Profile: Iflaviridae. J. Gen. Virol..

[35] Calla B., Hall B., Hou S., Geib S.M. (2014). A genomic perspective to assessing quality of mass-reared SIT flies used in Mediterranean fruit fly (Ceratitis capitata) eradication in California. BMC Genom..

[36] Webster C.L., Longdon B., Lewis S.H., Obbard D.J. (2016). Twenty-Five New Viruses Associated with the Drosophilidae (Diptera). Evol. Bioinform..

[37] Sharpe S.R., Morrow J.L., Brettell L.E., Shearman D.C., Gilchrist S., Cook J.M., Riegler M. (2021). Tephritid fruit flies have a large diversity of co-occurring RNA viruses. J. Invertebr. Pathol..

[38] Remnant E.J., Baty J.W., Bulgarella M., Dobelmann J., Quinn O., Gruber M.A.M., Lester P.J. (2021). A Diverse Viral Community from Predatory Wasps in Their Native and Invaded Range, with a New Virus Infectious to Honey Bees. Viruses.

[39] Ju H., Lim H., Domier L.L. (2020). Soybean Thrips (Thysanoptera: Thripidae) Harbor Highly Diverse Populations of Arthropod, Fungal and Plant Viruses. Viruses.

[40] Webster C.L., Waldron F.M., Robertson S., Crowson D., Ferrari G., Quintana J.F., Brouqui J.M., Bayne E.H., Longdon B., Buck A.H. (2015). The discovery, distribution, and evolution of viruses associated with Drosophila melanogaster. PLoS Biol..

[41] Medd N.C., Fellous S., Waldron F.M., Xue A., Nakai M., Cross J.V., Obbard D.J. (2018). The virome of Drosophila suzukii, an invasive pest of soft fruit. Virus Evol..

[42] Mahar J.E., Shi M., Hall R.N., Strive T., Holmesa E.C. (2020). Comparative Analysis of RNA Virome Composition in Rabbits and Associated Ectoparasites. J. Virol..

[43] Dalmon A., Gayral P., Decante D., Klopp C., Bigot D., Thomasson M., Herniou E.A., Alaux C., Conte Y. (2019). Le Viruses in the Invasive Hornet Vespa velutina. Viruses.

[44] Longdon B., Murray G.G.R., Palmer W.J., Day J.P., Parker D.J., Welch J.J., Obbard D.J., Jiggins F.M. (2015). The evolution, diversity, and host associations of rhabdoviruses. Virus Evol..

[45] King A.M.Q., Adams M.J., Carstens E.B., Lefkowitz E.J. (2011). Family Reoviridae. Virus Taxonomy: Ninth Report of the International Committee on Taxonomy of Viruses.

[46] Dedkov V.G., Dolgova A.S., Safonova M.V., Samoilov A.E., Belova O.A., Kholodilov I.S., Matsvay A.D., Speranskaya A.S., Khafizov K., Karganova G.G. (2021). Isolation and characterization of Wad Medani virus obtained in the tuva Republic of Russia. Ticks Tick. Borne. Dis..

[47] Qin X.-C., Shi M., Tian J.-H., Lin X.-D., Gao D.-Y., He J.-R., Wang J.-B., Li C.-X., Kang Y.-J., Yu B. (2014). A tick-borne segmented RNA virus contains genome segments derived from unsegmented viral ancestors. Proc. Natl. Acad. Sci. USA.

[48] Jia N., Liu H.B., Ni X.B., Bell-Sakyi L., Zheng Y.C., Song J.L., Li J., Jiang B.G., Wang Q., Sun Y. (2019). Emergence of human infection with Jingmen tick virus in China: A retrospective study. EBioMedicine.

[49] Kholodilov I.S., Belova O.A., Morozkin E.S., Litov A.G., Ivannikova A.Y., Makenov M.T., Shchetinin A.M., Aibulatov S.V., Bazarova G.K., Bell-sakyi L. (2021). Geographical and Tick-Dependent Distribution of Flavi-Like Alongshan and Yanggou Tick Viruses in Russia. Viruses.

[50] Kholodilov I.S., Litov A.G., Klimentov A.S., Belova O.A., Polienko A.E., Nikitin N.A., Shchetinin A.M., Ivannikova A.Y., Bell-sakyi L., Yakovlev A.S. (2020). Isolation and Characterisation of Alongshan Virus in Russia. Viruses.

[51] Moureau G., Cook S., Lemey P., Nougairede A., Forrester L., Khasnatinov M., Charrel R.N., Firth A.E., Gould E.A., de Lamballerie X. (2015). New Insights into Flavivirus Evolution, Taxonomy and Biogeographic History, Extended by Analysis of Canonical and Alternative Coding Sequences. PLoS ONE.

[52] Karabatos N. (1985). International Catalogue of Arboviruses.

[53] Longdon B., Wilfert L., Obbard D.J., Jiggins F.M. (2011). Rhabdoviruses in Two Species of Drosophila: Vertical Transmission and a Recent Sweep. Gebetics.

[54] Aznar-lopez C., Vazquez-moron S., Marston D.A., Juste J., Iba C., Berciano J.M., Salsamendi E., Aihartza J., Banyard A.C., Mcelhinney L. (2013). Detection of rhabdovirus viral RNA in oropharyngeal swabs and ectoparasites of Spanish bats. J. Gen. Virol..

