# Supplementary material for: Possible Arbovirus Found in Virome of Melophagus ovinus
Source: Viruses. 2021 Nov 26;13(12):2375. doi: 10.3390/v13122375 (PMC8707155; doi:10.3390/v13122375)
Supplement: Supplementary file 1 [file viruses-13-02375-s001.zip › viruses-1481119-supplementary/s1.pdf]

Table S1. Oligonucleotides used for the virus detection

Virus	Primer set	Oligonucleotide	Amplicon length	Temperature
BKUMSV	LL_1st_f	5`-aggcgatttggaggaaaggt-3`	980	50 °C
	LL_1st_r	5`-tcatacaaccattggtcgtaact-3`		
UMSV	hdw_1st_f	5`-cagagctgtgatggatatgt-3`	968	50 °C
	hdw_1st_r	5`-aacatctctcttactaacgac-3`		
ADMSV	ASDV_test_F	5`-ccagcaagtattcaaattgacaca-3`	573	50 °C
	ASDV_test_R	5`-gcaggattgagagatttgtc-3`		
KMIV	KILV_test_F	5`-tgtagagttgttattggtaaacttag-3`	471	50 °C
	KILV_test_R	5`-tcgaatcatttcattccattc-3`		
BBMRV	BBRV_test_F	5`-cgaatagaagaatgggaactga-3`	531	50 °C
	BBRV_test_R	5`-cagtgttttgagaatgtgtgc-3`		

Table S2. Estimates of evolutionary divergence between Ifla-like contigs found in the study.

	20_Ifla_N2	20_Ifla_N160_7_3_98_268	23_Ifla_N1	23_Ifla_N2_93	24_Ifla_N3	24_Ifla_N4_77
20_Ifla_N2						
20_Ifla_N160_7_3_98_268	0.0888					
23_Ifla_N1	0.0648	0.0907				
23_Ifla_N2_93	0.0893	0.0906	0.0884			
24_Ifla_N3	0.0893	0.0906	0.0884	0.0002		
24_Ifla_N4_77	0.0657	0.0919	0.0088	0.0903	0.0903	
22_Ifla_N140_2_12	0.0947	0.0922	0.0951	0.0912	0.0912	0.0984

The number of base substitutions per site from between sequences are shown. Analyses were conducted using the Maximum Composite Likelihood model [1]. This analysis involved 4 nucleotide sequences. All ambiguous positions were removed for each sequence pair (pairwise deletion option). Evolutionary analyses were conducted in MEGA X [2]

Table S3. Estimates of evolutionary divergence between sigmavirus-like contigs found in the study

	20-WLF-like	21_WLF_N1	21-WLF-like_N2_N3	22_WLF-like	23_WLF	24_WLF_N1
20-WLF-like						
21_WLF_N1	0,00679					
21-WLF-like_N2_N3	0,05072	0,05064				
22_WLF-like	0,00762	0,00817	0,05052			
23_WLF	0,00773	0,00810	0,05016	0,00505		
24_WLF_N1	0,00956	0,00994	0,05213	0,00504	0,00201	
24_WLF_N2	0,00947	0,00984	0,05223	0,00568	0,00211	0,00119

The number of base substitutions per site from between sequences are shown. Analyses were conducted using the Maximum Composite Likelihood model [1]. This analysis involved 4 nucleotide sequences. All ambiguous positions were removed for each sequence pair (pairwise deletion option). Evolutionary analyses were conducted in MEGA X [2]

Table S4. Estimates of evolutionary divergence between reo-like contigs and closes relative for each segment found in the GenBank database

	Estimates of evolutionary divergence between pool 23 and pool 24	Amino acid identity to the closest GenBank entry	Closest GenBank entry
Segment 1	0.0005*	61%	Bloomfield virus segment 1 (MF416371.1)
Segment 2	0.0072	55%	Bloomfield virus segment 2(KP714091.1)
Segment 3	0.0011	36%	Hubei odonate virus 14 (KX884685.1)
Segment 4	0.0049	40%	Bloomfield virus segment 4 (KP714092.1)
Segment 5	0.0037	35%	Bloomfield virus segment 5 (KP714093.1)
Segment 6	0.0215	49%	Bloomfield virus segment 6 (KP714094.1)
Segment 7	0.0148	61%	Bloomfield virus segment 7 (KP714095.1)
Segment 8	0.0044	28%	Bloomfield virus segment 8 (KP714096.1)
Segment 9	0.0019	44%	Bloomfield virus segment 9 (KP714097.1)
Segment 10	0.0290	33%	Bloomfield virus segment 10 (KP714098.1)

* - The number of base substitutions per site from between sequences are shown. Analyses were conducted using the Maximum Composite Likelihood model [1]. This analysis involved 4 nucleotide sequences. All ambiguous positions were removed for each sequence pair (pairwise deletion option). Evolutionary analyses were conducted in MEGA X [2]

Figure S1. Electrophoresis of ADMSV PCR-detection in the ked suspensions. Ked suspension numbers are shown. “K-“ - negative control (water added instead of cDNA). Blue line corresponds to the expected length of the PCR-positive probe.

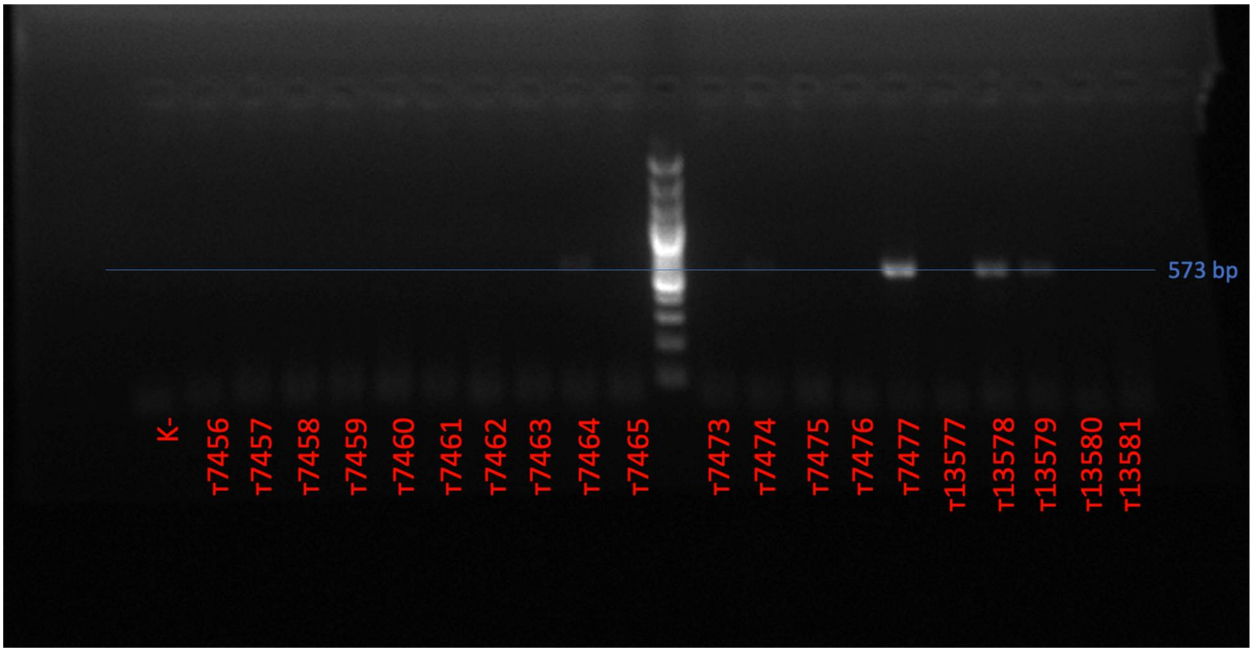

Figure S2. Electrophoresis of ADMSV PCR-detection after the first passage in the PEK cells. Ked suspension numbers are shown. “K-“ - negative control (water added instead of cDNA). “K+“ - positive control. Blue line corresponds to the expected length of the PCR-positive probe.

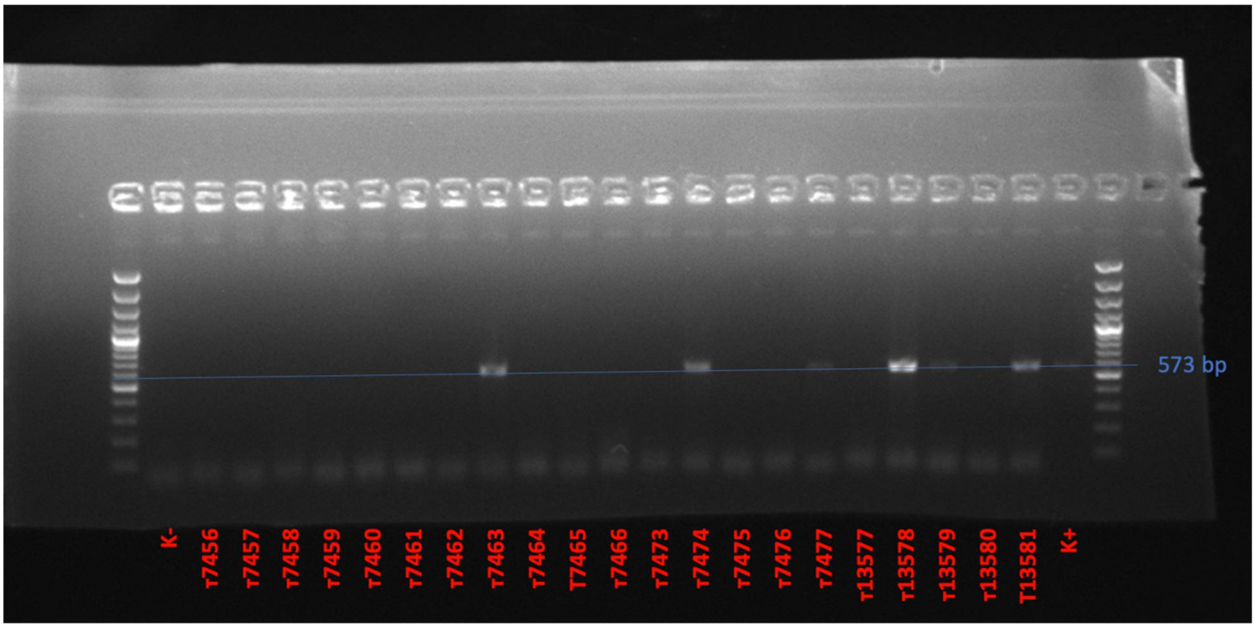

Figure S3. Electrophoresis of ADMSV PCR-detection after the second passage in the PEK cells. Ked suspension numbers are shown. “K-“ - negative control (water added instead of cDNA). Blue line corresponds to the expected length of the PCR-positive probe.

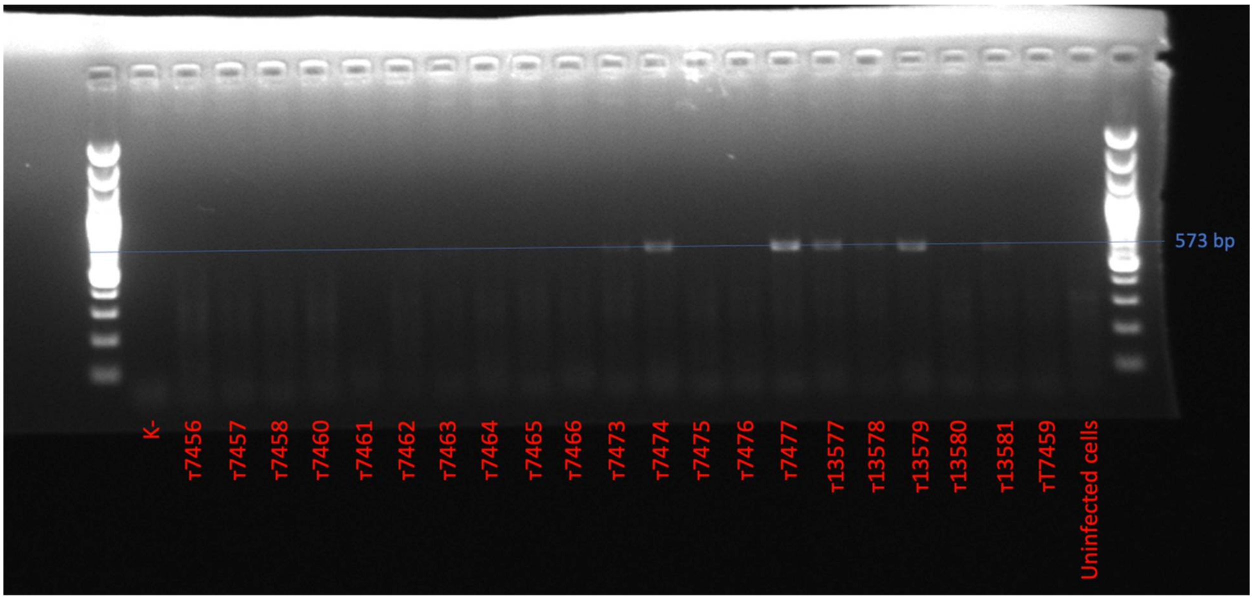

Figure S4. Electrophoresis of ADMSV PCR-detection after the third passage in the PEK cells. Ked suspension numbers are shown. “K-“ - negative control (water added instead of cDNA). “K+“ - positive control. Blue line corresponds to the expected length of the PCR-positive probe.

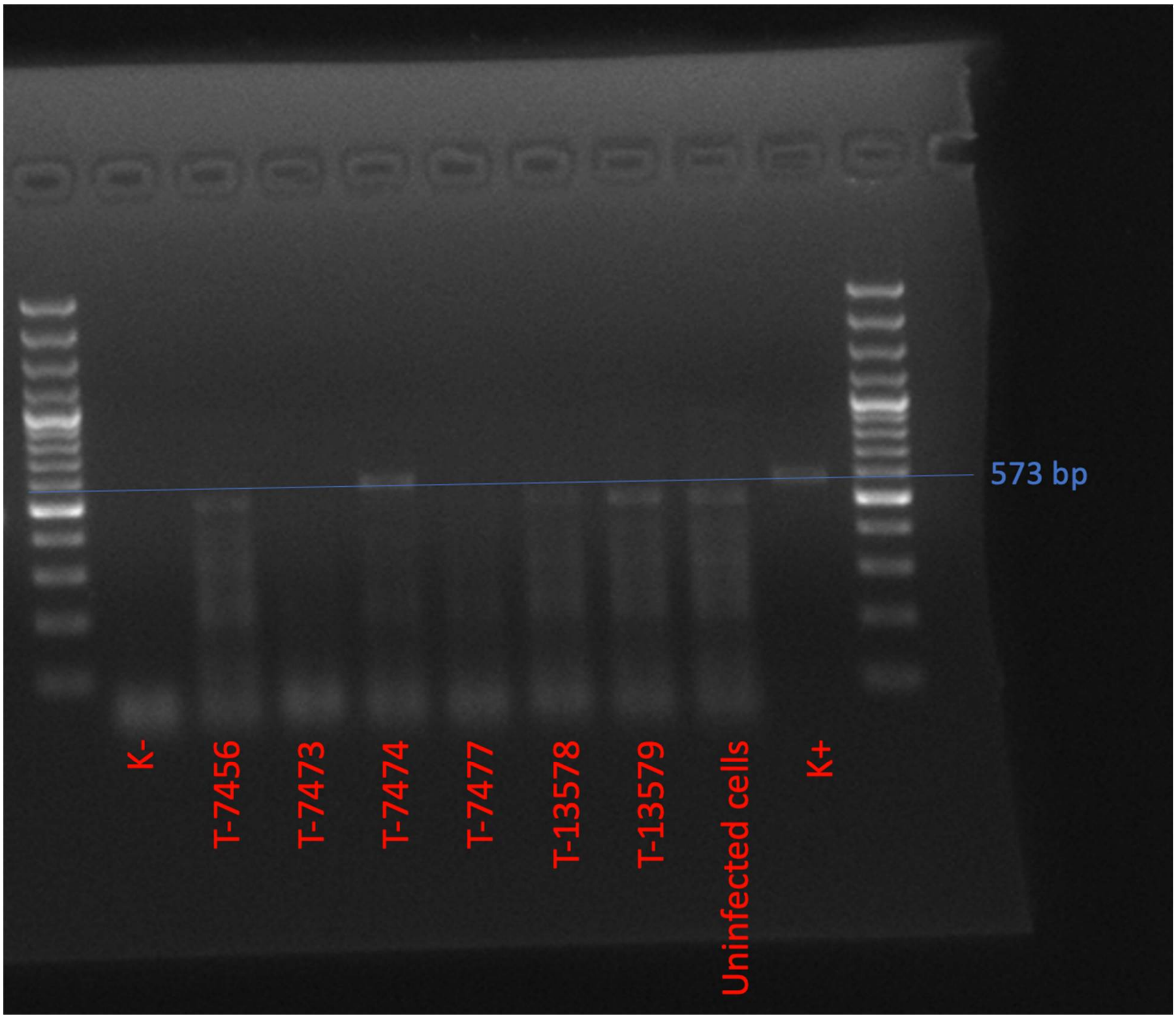

1. Tamura, K.; Nei, M.; Kumar, S. Prospects for inferring very large phylogenies by using the neighbor-joining method. In *Proceedings of the National Academy of Sciences (US*A), July 2004; pp. 101:11030–11035.

2. Kumar, S.; Stecher, G.; Li, M.; Knyaz, C.; Tamura K. MEGA X: Molecular Evolutionary Genetics Analysis across computing platforms. *Mol. Biol. Evol.* **2018,** *35*, 1547–1549.
